# Supramolecular Structures of the *Dictyostelium* Lamin NE81

**DOI:** 10.3390/cells8020162

**Published:** 2019-02-16

**Authors:** Marianne Grafe, Petros Batsios, Irene Meyer, Daria Lisin, Otto Baumann, Martin W. Goldberg, Ralph Gräf

**Affiliations:** 1 Department of Cell Biology, University of Potsdam, Karl-Liebknecht-Str. 24-25, 14476 Potsdam-Golm, Germany; mgrafe@uni-potsdam.de (M.G.); irene.meyer@uni-potsdam.de (I.M.); d.lisin@web.de (D.L.); 2 Department of Animal Physiology, University of Potsdam, Karl-Liebknecht-Str. 24-25, 14476 Potsdam-Golm, Germany; obaumann@uni-potsdam.de; 3 Department of Biosciences, Durham University, Science Laboratories, South Road, Durham DH1 3LE, UK; m.w.goldberg@durham.ac.uk

**Keywords:** lamin, NE81, *Dictyostelium*, nuclear envelope, nuclear lamina, expansion microscopy

## Abstract

Nuclear lamins are nucleus-specific intermediate filaments (IF) found at the inner nuclear membrane (INM) of the nuclear envelope (NE). Together with nuclear envelope transmembrane proteins, they form the nuclear lamina and are crucial for gene regulation and mechanical robustness of the nucleus and the whole cell. Recently, we characterized *Dictyostelium* NE81 as an evolutionarily conserved lamin-like protein, both on the sequence and functional level. Here, we show on the structural level that the *Dictyostelium* NE81 is also capable of assembling into filaments, just as metazoan lamin filament assemblies. Using field-emission scanning electron microscopy, we show that NE81 expressed in *Xenopous* oocytes forms filamentous structures with an overall appearance highly reminiscent of *Xenopus* lamin B2. The in vitro assembly properties of recombinant His-tagged NE81 purified from *Dictyostelium* extracts are very similar to those of metazoan lamins. Super-resolution stimulated emission depletion (STED) and expansion microscopy (ExM), as well as transmission electron microscopy of negatively stained purified NE81, demonstrated its capability of forming filamentous structures under low-ionic-strength conditions. These results recommend *Dictyostelium* as a non-mammalian model organism with a well-characterized nuclear envelope involving all relevant protein components known in animal cells.

## 1. Introduction

In all eukaryotes, the nuclear envelope consists of an outer and inner membrane. The outer nuclear membrane (ONM) is directly connected both to the endoplasmic reticulum and, at the nuclear pore complexes (NPCs), to the inner nuclear membrane (INM). The perinuclear space separates the INM and ONM and is continuous with the lumen of the endoplasmic reticulum (ER). At the INM, transmembrane proteins and associated filamentous proteins within the nuclear matrix form a fibrous nuclear lamina. While higher plants (Archaeplastida) and some unicellular Excavata such as *Trypanosoma* employ specialized filamentous proteins to form fibrous protein assemblies at the INM, the major components of the nuclear lamina in metazoans are specialized intermediate filament (IF) proteins called lamins [1,2]. Through so-called linker of nucleoskeleton and cytoskeleton (LINC) complexes spanning both nuclear membranes [3], lamins and, hence, the nuclear lamina are indirectly connected with all cytosolic cytoskeletal elements. In addition, lamins associate with chromatin and are involved in the formation of lamina-associated heterochromatin domains. Thus, they also regulate epigenetic gene regulation and differentiation [4]. Due to the various binding activities of lamins, in particular to cytoskeletal elements, the nucleus serves also as an abutment against mechanical forces for the whole cell [5]. Lamin mutations affecting preprotein processing, disruptions of the lamin network, or its interactions with LINC complexes cause various devastating diseases called laminopathies [6]. These include Hutchinson–Gilford progeria syndrome (HGPS), Emery–Dreyfuss muscular dystrophy (EDMD), Charcot–Marie–Tooth disease (CMT), dilated cardiomyopathy (DCM), and several others [7]. In part, the pathogenic alterations in tissues affected by these diseases can be explained by a role of lamins in epigenetic gene regulation. However, the striking affection of tissues under mechanical stress (e.g., blood vessels, muscle, skin) emphasizes the importance of lamins in mechanobiology [8,9]. Thus, the etiology of these diseases cannot be understood without a profound knowledge of the supramolecular structures formed by lamins at the nuclear envelope. Although these structures were investigated since the 1980s of the last century, there is still no common scheme. In various cell types and organisms, lamins may assemble into filaments of variable thickness and spatial organization (see Section 4, as well as Reference [10] for a review). 

Lamins are found in all metazoans, even in organisms possessing no cytoplasmic IFs. Thus, they are considered the most ancient form of IFs [11]. For a long time, no lamins could be identified in bikonta, plants, fungi, and amoebozoans. Yet, we showed that the nuclear lamina of the model organism *Dictyostelium discoideum* contains a protein, NE81, that is not only evolutionarily related to lamins, but also performs major lamin functions [12,13]. The finding of a lamin in the eukaryotic supergroup Amoebozoa facilitated the identification of lamin-like proteins also in other eukaryotic clades previously thought to contain no lamins [14,15,16]. Through bioinformatics, homologs of metazoan lamins were meanwhile identified in most eukaryotic groups, i.e., in Opisthokonta including Choanoflagellata, Filasteria, and Ichtyosporea, in Amoebozoa, and in Dinoflagellata, Rhizaria, and Stramelopila of the SAR (Stramenopile, Alveolata, Rhizaria) group [16]. Thus, it is very likely that lamin-related proteins were already part of the molecular toolbox of the last eukaryotic common ancestor (LECA) [17]. 

Like all lamins, NE81 consists of an α-helical, central rod domain (370 amino acids (aa)) flanked by head and tail domains. The head domain includes a consensus sequence for phosphorylation by cyclin-dependent kinase 1 (CDK1) at position 122, while the tail domain is characterized by a nuclear localization sequence (NLS) at the beginning, a conserved lamin tail domain (LTD), and a CaaX-box (cysteine, two aliphatic aa, and X = residue specifying the type of isoprene moiety) for prenylation at the C-terminal end [16]. Our previous studies revealed that NE81 behaves like a lamin also on the functional level, i.e., it requires an intact CaaX box for proper INM association, it is required for centrosome–nucleus attachment and chromatin organization, and is essential for the mechanical robustness of the whole cell [12,13]. Our results suggested that NE81 is tethered to the INM through its prenyl anchor and assembles along the INM in a two-dimensional fashion, as proposed for B-type lamins. Disruption of CaaX box function caused three-dimensional assembly of GFP-tagged NE81 (GFP-NE81ΔCLIM) at the INM. GFP-NE81ΔCLIM clusters underwent cell-cycle-dependent assembly/disassembly. Point mutation of the CDK1 phosphorylation site at position 122 to prevent CDK1 phosphorylation abrogated this dynamic behavior and prevented disassembly at the onset of mitosis. In strains expressing GFP-NE81 without a functional NLS and CaaX-box (GFP-NE81ΔNLSΔCLIM), such clusters were found in the cytosol [18]. As GFP-NE81ΔCLIM clusters in the nucleus, they disappeared at the gap 2/mitosis (G2/M) transition and reappeared late in mitosis. Thus, they represented reversible three-dimensional protein assemblies. In electron microscopy (EM) images, NE81 clusters exhibited a spongy appearance and were uniformly studded with ribosomes, indicating co-translational assembly of GFP-NE81ΔNLSΔCLIM. In animals, importin-α effectively inhibits premature cytosolic assembly of lamins prior to nuclear import [19]. Thus, co-translational formation of NE81 clusters may also be due to the inability of importin-α to bind the mutated NLS. Our results clearly demonstrated that clusters formed by CaaX-box-deficient NE81 variants represented reversible three-dimensional protein assemblies, not misfolded protein aggregates. In this work, we show that these NE81 variants also provide a convenient source for native, assembly-competent NE81 to pursue in vitro studies of supramolecular NE81 assemblies. 

## 2. Materials and Methods

### 2.1. Vector Constructions 

NE81 coding sequences were cloned into expression vectors based on the N-terminal GFP-fusion vectors pIS76 and pIS77 (blasticidin and G418 resistance, respectively) as described previously [20]. In these vectors, protein expression is driven by the actin-6 promoter. For 8×HisMyc-vectors, the GFP cassette was replaced by the following oligo cassette: 5′ CATCAT CATCATCATCATCATCAT GCTGAAGAACAAAAATTAATTTCAGAAGAAGATTTA 3′, via the *NheI* and *SalI* restrictions sites. The HisMyc-NE81ΔNLSΔCLIM-S122E vector was generated by overlap extension PCR after amplification of two genomic NE81 DNA fragments, fragment 1 with B91 (5′ GTTGAGCTG CTCTATTTGGTtcTAATGGTG 3′) and a suitable forward *SalI*-linker primer, and fragment 2 with B89 (5′ CACAAATAGGTACACCATTAgaACCAAATAG 3′) and the suitable reverse *BamHI*-linker primer. CaaX-box and NLS deletions and mutations were performed by PCR using suitable linker primers containing *BamHI*/*SalI* restriction sites as described earlier [18]. For knockouts of endogenous NE81, we transformed the knockout vector described by Krüger et. al. [12] into strains expressing GFP-NE81 (full length) and HisMyc-NE81 (full length) driven by the actin-6 promoter. Successful insertion was confirmed by the absence of endogenous NE81 in Western blots and by PCR of genomic DNA. For construction of the FLAG-NE81 vector for expression in *Xenopus* oocytes, the complete NE81 sequence was custom synthesized, whereby each codon was optimized for expression in vertebrate systems using the GeneOptimizer technology (GenArt, Thermo Fisher Scientific, Waltham, MA, USA). The codon-optimized NE81 sequence flanked by *XhoI* and *XbaI* restriction sites was cloned into pCS2+ [21] yielding the plasmid pPB55.

### 2.2. Protein Purifications

*Dictyostelium* cells were grown, washed, and lysed as described before [22]. Cells were grown in 400 mL of HL5C medium with G418 up to 1 × 10^9^ cells. After washing with phosphate buffer, cells were resuspended with an equal volume of lysis buffer (50 mM Tris/Cl, pH = 8.0, 1 M NaCl, 20 mM imidiazole), protease inhibitor cocktail [23], and 1 mM dithiothreitol DTT. After lysis by filtration through 5-µm Nuclepore polycarbonate filters (Whatman, GE Healthcare, Freiburg, Germany), the cell suspension was centrifuged at 4000× *g* for 15 min at 4 °C. Supernatant was loaded directly onto Ni-NTA beads (Takara Bio Europe SAS, Saint-Germain-en-Laye, France) equilibrated with equilibration buffer (25 mM Tris/Cl, pH = 8.0, 0.5 M NaCl, 10 mM imidiazole). After washing with equilibration buffer and washing buffer (equilibration buffer with 25 mM imidiazole), the bound protein was eluted with elution buffer (25 mM Tris/Cl, pH = 8.0, 0.5 M NaCl, 250 mM imidiazole). Protein fractions were checked for protein expression by Coomassie-stained SDS-PAGE. Peak fractions were pooled and dialyzed overnight in 25 mM Tris/Cl, pH = 8.0, 0.5 M NaCl. The typical yield was ~1.5 mg of protein in 1.5 mL (measured by the Amido black assay, or absorption at 280 nm). The protein solution was briefly centrifuged at 17,000× *g*, and the supernatant was used for further analysis.

### 2.3. Assembly Studies

For electrophoretic analysis of assembly behavior, the purified protein was diluted with high-salt buffer (25 mM Tris/Cl, pH = 8.0, 0.5 M NaCl, 1 mM DTT) to the indicated concentration and dialyzed (SpectraPor dialysis tubing, Repligen, Ravensburg, Germany, 3.5-kDa cut-off membrane) for 4 h at 4 °C into the respective low-salt buffers (25 mM Tris/Cl, pH = 8.0, 1 mM DTT, various NaCl concentrations) followed by centrifugation (17,000× *g*, 1 h, 4 °C). The pellets were resuspended in a buffer volume equal to the supernatants, and 12 µL of each sample containing 2 µL of 6x Laemmli buffer was loaded on SDS-PAGE followed by Western blotting. Densitometric measurements of immunoblot bands were performed with ImageJ software [24]. 

For light microscopic assembly studies, the purified protein was diluted with high-salt buffer and dialyzed as described above. Then, 10–25 µg protein was centrifuged onto round 12-mm coverslips (4500× *g*, 10 min, 4 °C), followed by incubation in fixative (3.7% formalin in phosphate or Tris-buffered saline, pH = 8.0) for 5 min.

### 2.4. Light Microscopy

Cells were fixed on coverslips for 5 min with glutaraldehyde as described earlier [25]. Wide-field fluorescence microscopy was performed as described previously [26] using a Zeiss CellObserver HS system equipped with a PlanApo 1.4/100× objective, an Axiocam MRm Rev.3 charge-coupled device (CCD) camera and a piezo stage (Carl Zeiss Mikroskopie GmbH, Jena, Germany). For deconvolution of image stacks of fixed cells, the iterative algorithm of Axiovision 4.8 (Carl Zeiss Mikroskopie GmbH, Jena, Germany) and either a point spread function (PSF) measured with 200-nm Tetraspeck beads (conventional light microscopy; Thermo Fisher Scientific, Waltham, MA, USA) or a theoretical PSF (expansion microscopy) was employed. 

Stimulated emission depletion (STED) microscopy was carried out essentially as described previously using a retrofitted confocal microscope based on an Olympus inverted time-resolved confocal scanning microscope (MicroTime 200, PicoQuant, Berlin, Germany) equipped with an Olympus UPLSAPO 1.4/100× objective [27,28].

Expansion microscopy (ExM) was carried out according to Tillberg et al. and Chozinski et al. [29,30]. Samples were prepared as described for standard light microscopy; however, incubation times for primary (up to 8h) and secondary antibodies (up to 4 h) were increased, and antibodies were used at a twofold higher concentration. After staining, samples were post-fixed for 10 min with 0.25% glutaraldehyde. Gelation of polyacrylamide took place for 30 min at 37 °C in a self-made slide chamber with coverslips (type #4; Menzel-Gläser, Braunschweig, Germany) as a spacer. After proteinase K digestion for 45 min at 37 °C, the gels were expanded for two hours in water followed by a 30-min incubation with Hoechst 33342 (50 µg/mL in water) for chromatin staining. For imaging, we used the same Zeiss CellObserver HS system equipped with a PlanApo 1.3/63 objective.

### 2.5. Transmission Electron Microscopy (TEM)

TEM was performed with a Philips CM100 electron microscope (FEI Deutschland GmbH, Frankfurt/Main, Germany). For ultrathin sectioning, *Dictyostelium* cells were fixed and embedded as described previously [18]. For negative staining, 4-µL aliquots of the dialyzed protein solution were adsorbed for 2 min to pioloform-coated copper grids, fixed for 2 min with 2.5% glutaraldehyde in assembly buffer and stained with 1% uranyl acetate.

### 2.6. Field-Emission Scanning Electron Microscopy (feSEM) of Xenopus Oocyte Nuclear Membranes

*Xenopus* oocyte isolation and microinjection was performed essentially as described previously [31]. Female *X. laevis* organisms were purchased from NASCO (Fort Atkinson, WI, USA). Oocytes were surgically removed and were defolliculated by collagenase treatment, as described previously [32]. Plasmid DNA (27.5 to 110 ng/µL in water) was injected into the oocyte nucleus (13.8 nL per nucleus) with a Nanoliter-Injector (World Precision Instruments, Sarasota, FL, USA). DNA was mixed with Blue Dextran (10 mg/mL final concentration) (Fluka, München, Germany) to confirm successful nuclear injection. Synthetic RNA (440 ng/µL in water) was injected into the cytoplasm of oocytes (13.8 to 27.6 nL per oocyte). Injected oocytes were incubated for 16 to 24 h at 18 °C to allow expression of proteins. Preparation of *Xenopus* oocyte nuclear membranes was performed according to Reference [33]. Oocytes were washed briefly and placed in 5:1 buffer (17 mM NaCl, 83 mM KCl, 10 mM HEPES/KOH pH 7.4). Under a stereo dissecting microscope, a sharpened dissecting needle was used to pierce the animal pole of the oocyte and the nucleus was extruded. Nuclei were transferred to a silicon chip (Agar Scientific, Stansted, UK), allowed to adhere, and the nuclear envelope was spread with fine glass needles. Chips were then fixed in 2% glutaraldehyde, 0.2% tannic acid, 0.1 M HEPES/KOH pH 7.4 for at least 10 min, washed briefly two times in deionized water, then placed in 0.1% OsO_4_ for 10 min, washed briefly in water, and dehydrated through an ethanol series, and critical-point dried (Baltec CPD 030). Samples were then sputter-coated with 1–2 nm of chromium (Cressington, UK, model 328) and viewed in a Hitachi S-5200 field-emission scanning electron microscope at 10-kV accelerating voltage. 

### 2.7. Other Methods

*Dictyostelium* cells (strain AX2) were cultured in HL5c medium (Formedium, Hunsanton, UK) at 21 °C supplemented with sterile-filtered glucose added after autoclaving, either adherently in tissue culture flasks for transformation by electroporation [34], or in suspension in Erlenmeyer flasks on a rotary shaker at 150 rpm for protein expression. SDS electrophoresis and Western blotting with either nitro-blue tetrazolium and 5-bromo-4-chloro-3′-indolyphosphate (NBT/BCIP) color detection or enhanced chemiluminescence was performed as described earlier [23].

### 2.8. Antibodies and Conjugates

Primary antibodies used were polyclonal rabbit anti-NE81 [12], monoclonal rat YL1/2 directed against α-tubulin [35], monoclonal mouse 9E10 directed against myc-tag (EQKLISEEDL) [36], and monoclonal mouse Act-1 against *Dictyostelium* actin [37]. Secondary antibodies involved Alexa Fluor conjugates purchased from Life Technologies (Darmstadt, Germany), Atto conjugates from Atto-Tec GmbH (Siegen, Germany), and enzyme conjugates for Western blotting from Sigma (Deisenhofen, Germany).

## 3. Results

For our structural analysis of NE81, we pursued two strategies. Firstly, we used the *Xenopus* system to obtain NE81 assemblies at *Xenopus* oocyte nuclear membranes for scanning electron microscopy (feSEM), as successfully performed by Goldberg and co-workers [31]. Secondly, we opted for expression of tagged NE81 in *Dictyostelium* cells followed by affinity purification and in vitro assembly.

### 3.1. Field-Emission SEM Analysis of NE81 Assemblies at Xenopus Oocyte Nuclear Membranes

**Figure 1:**
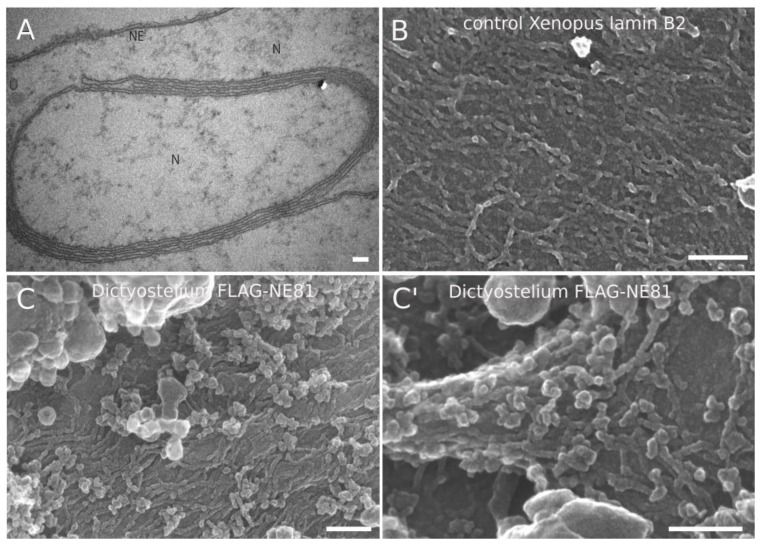
Filamentous structures at intranuclear membrane stacks obtained upon expression of FLAG-NE81 in *Xenopus* oocytes. (**A**) TEM image showing intranuclear membrane stacks elicited through FLAG-NE81 expression. (**B**,**C**,**C′**) Field-emission (fe)SEM image showing *Xenopus* lamin B2 filaments (**B**) and *Dictyostelium* FLAG-NE81 filaments (**C**,**C′**) associated with intranuclear membrane stacks. Scale bars = 100 nm.

Previous work proved the *Xenopus* oocyte expression system as a valuable tool to display lamin assemblies at the inner surface of the nuclear envelope, as the latter can be manually dissected and separated from chromatin, which is not tightly attached to the NE in these cells [38]. Here, expression of lipidated nuclear proteins carrying a CaaX-box such as lamins results in formation of intranuclear membrane stacks [31,39]. These membrane stacks turned out to be useful to analyze lamin filaments other than *Xenopus* lamin LIII [31]. Here, we expressed codon-optimized, FLAG-tagged NE81 using a vertebrate expression vector, and analyzed the oocyte NE by electron microscopy. TEM images showed the stacking of intranuclear membranes, which is typically observed upon expression of B-type lamins (Figure 1A). Field-emission SEM analysis revealed short filaments, some of which were oriented in a parallel fashion, but also globular aggregates (Figure 1C,C′). The overall appearance was very reminiscent of *Xenopus* lamin B2 (Figure 1B) in this assay, although NE81 filaments appeared shorter than those of lamin B2. NE81 filaments exhibited a diameter of ~8.5 ± 0.9 nm (mean ± SD; *n* = 50), i.e., slightly thicker than the published ~7.3 ± 0.9 nm of lamin B2 filaments in the same assay (Figure 1B), but thinner than the 11.7 ± 1.2 of lamin LIII [31]. Regardless of their size, the most important conclusion from this assay is that heterologously expressed NE81 is indeed capable of assembling into filamentous structures highly reminiscent of metazoan lamin assemblies.

### 3.2. In Vitro Assembly of NE81 Expressed in Dictyostelium

#### 3.2.1. NE81 Lacking CaaX-box and NLS Is a Suitable Source to Study Protein Assembly

For further in vitro studies, we looked for a convenient and suitable source of recombinant NE81. In analogy to successful experiments in the *Caenorhabditis elegans* system, we initially expected bacterially expressed NE81 as a suitable starting material for assembly/disassembly experiments [40]. 

**Figure 2:**
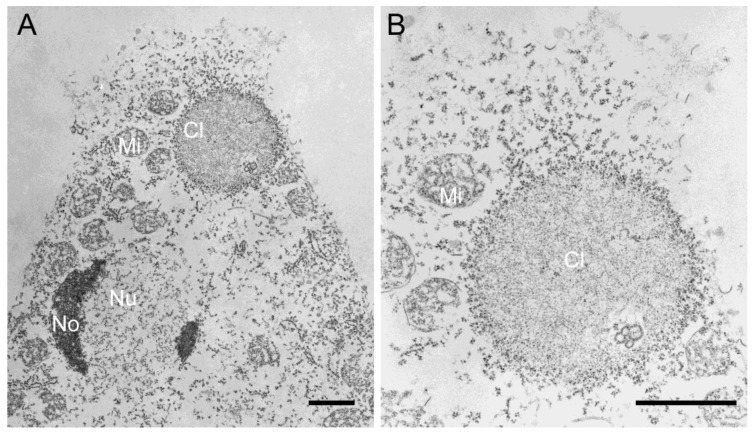
(**A**,**B**) Transmission electron microscopy of permeabilized *Dictyostelium* cells showing spongy GFP-NE81ΔNLSΔCLIM clusters (Cl) studded by particles representing ribosomes. The nucleus (Nu), nucleoli (No), and mitochondria (Mi) are labeled. (**B**) is an enlarged view of (**A**). Scale bars = 1 µm.

Thus, we firstly tried purified MBP-NE81 that was also used to generate our anti-NE81 antiserum [12]. Yet, in the course of our experiments, we found that the bacterially expressed protein had a tendency to precipitate upon storage. Although this behavior may reflect a slow assembly of the protein in the purification buffer, it was more likely due to unspecific aggregation. We were unable to find a condition to keep the protein in a soluble state. Attempts to purify the recombinant protein under denaturing conditions also did not yield sufficient amounts of intact, soluble protein. Expression of untagged NE81 in *Escherichia coli* for the production of inclusion bodies, an established strategy for animal lamins [41], failed due to a low expression level. Taken together, we concluded that bacterial expression is not a suitable method to obtain sufficient amounts of NE81 in a functional, correctly folded state. Therefore, we switched to *Dictyostelium* as the autologous expression system. Our previous results clearly showed that clusters formed by CaaX-box-deficient NE81 variants represented three-dimensional protein assemblies, which disappeared at mitotic onset and reappeared in late telophase. Moreover, they showed a very homogeneous appearance with an intermediate electron density at the ultrastructural level (Figure 2) [12,18]. Due to these properties, these clusters, and especially the cytosolic ΔNLSΔCLIM variant, appeared as a useful source to isolate assembly-competent NE81 for in vitro experiments. This idea was supported by a pre-experiment, in which we analyzed the particular material within a cytosolic extract of GFP-NE81ΔNLSΔCLIM cells, and found that the green fluorescent protein clusters were still present (not shown).

#### 3.2.2. The GFP Tag, But Not the HisMyc-Tag, Interferes with NE81 Protein Assembly

**Figure 3:**
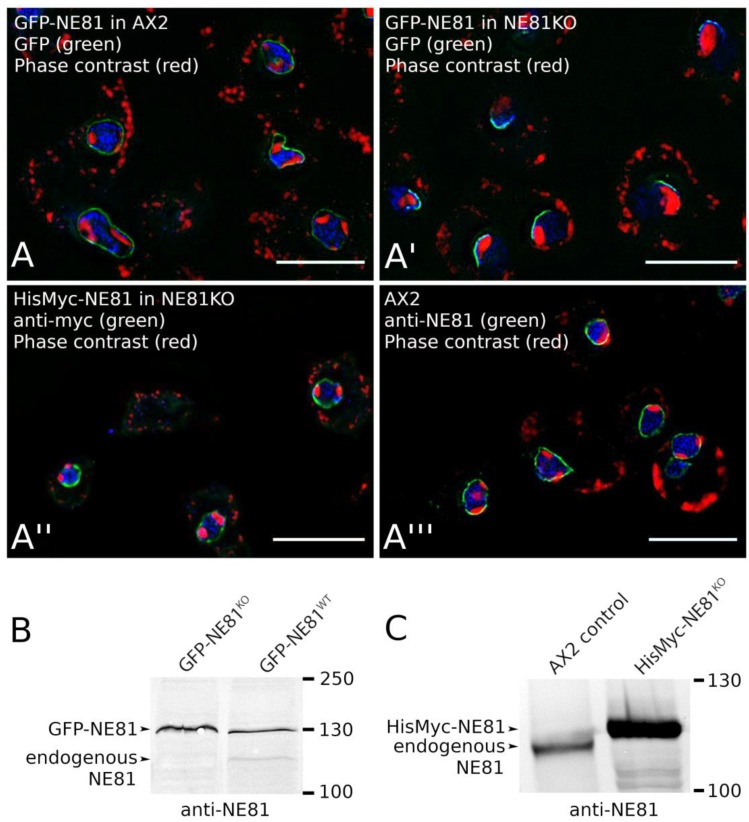
Expression of tagged NE81 in AX2 control cells or NE81 knockout cells. (**A**) Fluorescence deconvolution microscopy using a PlanApo 1.4/100× objective; GFP-NE81 cells (**A**), GFP-NE81^KO^ cells (**A′**), HisMyc-NE81^KO^ cells (**A′′**), and AX2 control cells (**A′′′**) were fixed with glutaraldehyde, stained with 4′,6-diamidino-2-phenylindole (DAPI) and, if appropriate (**A′′′**), with anti-Myc/anti-mouse-AlexaFluor 488 or anti-NE81/anti-rabbit-AlexaFluor 488. Scale bar = 5 µm. NE81 fluorescence is shown in green, DAPI in blue, and inverted phase contrast emphasizing the dark nucleoli is shown in red. (**B**,**C**) Immunoblots stained with alkaline phosphatase/NBT/BCIP show the absence of endogenous NE81 in the respective knockout (KO) strains and the band shift of the tagged protein compared to endogenous NE81.

Prior to using GFP as a protein purification tag, we set out to investigate whether the large N-terminal GFP-tag would interfere with NE81 assembly. This suspicion was fueled by earlier publications on human vimentin. When artificially expressed in the nucleus, human GFP-vimentin (containing an NLS) did not form filaments, while it became capable of doing so if co-expressed with untagged vimentin [42]. When expressed in the endogenous NE81 background in *Dictyostelium*, GFP-NE81 showed the same localizations as endogenous NE81 [12] (Figure 3A,A′′′). This strain was used to disrupt the endogenous *NE81* gene by homologous recombination, in order to force cells to live with the GFP-fusion protein only. The absence of endogenous NE81 protein was proven by Western blotting (Figure 3B). In this GFP-NE81 knockout (KO) strain, GFP-NE81 showed a crescent-like, pericentrosomal distribution, which was never observed in cells still expressing endogenous NE81 (Figure 3A,A′). Furthermore, the usual two to three nucleoli, nicely visible as perinuclear dark zones in phase-contrast images, were clustered into only one nucleolus associated with the NE81 crescent. Irrespective of the molecular causes, the unusual distribution of the GFP-NE81 fusion protein and the poor growth of this strain both indicated that GFP-NE81 was not fully functional, possibly due to a compromised ability to assemble properly. Thus, the use of GFP-NE81 and its variants as a source for NE81 isolations was abandoned and we switched to an N-terminal 8×His-Myc-tag to replace GFP. Firstly, we set out to show that this tag did not interfere with NE81 localization and function. In cells expressing HisMyc-NE81, we knocked out the *NE81* gene. Again, the absence of endogenous NE81 was confirmed by Western blotting (Figure 3C). The resulting HisMyc-NE81^KO^ strain exhibited a comparable growth rate to AX2 control cells, and showed an even distribution of HisMyc-NE81 at the nuclear envelope as holds true for endogenous NE81 in control cells (Figure 3A′′,A′′′). 

#### 3.2.3. Isolation of HisMyc-NE81ΔNLSΔCLIM and In Vitro Assembly of Filaments

**Figure 4:**
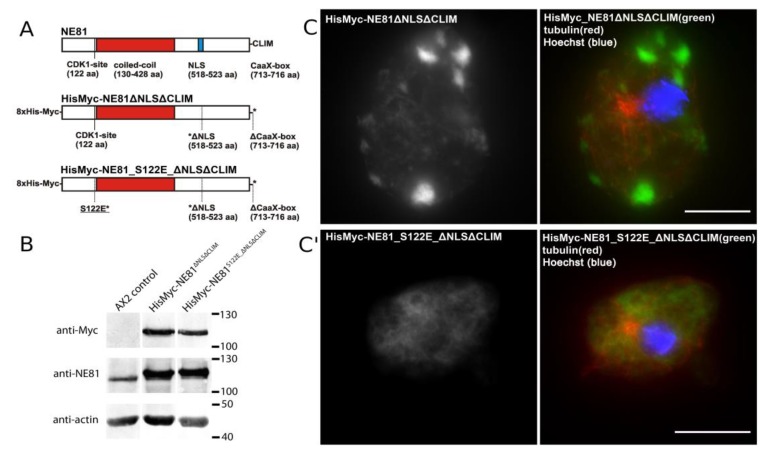
Formation of cytosolic HisMyc-NE81ΔNLSΔCLIM clusters requires an intact S122 target for CDK1. (**A**) Domain overview of HisMyc-NE81 variants used in this work. (**B**) Immunoblots of whole-cell extracts showing relative expression levels of endogenous NE81 compared to the tagged variants. The anti-actin staining is shown as a loading control. (**C**,**C′**) Expansion microscopy employing an LCI PlanNeo 1.3/63× objective. Cells were fixed with glutaraldehyde and stained with Hoechst33342, anti-myc/anti-mouse-AlexaFluor 488, and anti-tubulin/anti-rat-AlexaFluor 568 as indicated. Cytosolic HisMyc-NE81ΔNLSΔCLIM clusters are present only in cells with a native serine 122 (**C**), but not in cells carrying the phosphomimetic S122E mutation (**C′**). Expansion factors are 3.2 in (**C**) and 3.7 in (**C′**); scale bars = 5 µm (referring to the original size).

Preliminary experiments showed that cytosolic NE81 assemblies were easier to isolate than NE-associated assemblies contaminated with chromatin. We used a strain expressing HisMyc-NE81 with a non-functional NLS (replacement of basic residues by alanine) and a deleted CaaX-box (i.e., HisMyc-NE81ΔNLSΔCLIM; Figure 4A). In this strain, protein levels for HisMyc-NE81ΔNLSΔCLIM were higher compared to endogenous NE81 in control cells, while expression of endogenous NE in the same strain was slightly suppressed (Figure 4B). 

The phenomenon of suppressed endogenous proteins upon high expression levels of the corresponding tagged fusion protein is frequently observed in very different *Dictyostelium* overexpression strains [18,43]. As in the corresponding GFP-variant [18] HisMyc-NE81ΔNLSΔCLIM formed large, cell-cycle-dependent cytosolic protein clusters (Figure 4C). Clusters were absent in mitotic cells [18] and in HisMyc-NE81_S122EΔNLSΔCLIM cells where the CDK1 target serine 122 was replaced by a phosphomimetic glutamate point mutation (Figure 4C′). This is in line with other data indicating that the mitotic disassembly of NE81 protein clusters is triggered by CDK1 phosphorylation at serine 122 [12]. 

**Figure 5:**
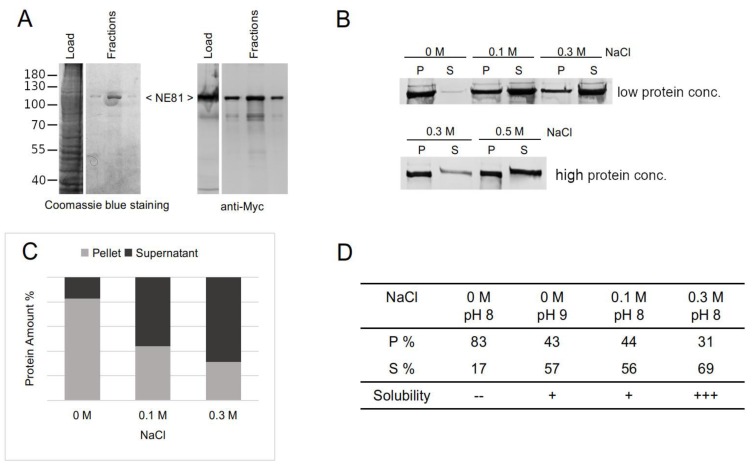
Formation of HisMyc-NE81ΔNLSΔCLIM assemblies is salt-dependent in vitro. (**A**) Affinity purification of HisMyc-NE81ΔNLSΔCLIM expressed in *Dictyostelium* at high-salt conditions (0.5 M NaCl). Proteins were separated on an 8% SDS-PAGE. A Coomassie blue staining (left) and a Western blot stained with anti-Myc/alkaline phosphatase and NBT/BCIP color detection of the total extract and Ni-NTA-chromatography fractions are shown. Molecular masses of standard proteins are indicated on the left. (**B**) Soluble purified HisMyc-NE81ΔNLSΔCLIM at two starting concentrations (low concentration = 0.2 mg/mL; high concentration = 0.4 mg/mL) was dialyzed against 25 mM Tris-HCl pH = 8.0 and indicated NaCl concentrations, followed by centrifugation. Equivalent amounts of pellet (P) and supernatant (S) were loaded on SDS gels, blotted, stained with anti-myc antibodies, and detected as described above. Low concentration: protein amount in the pellet decreases with increasing NaCl concentration. (**C**) Chart depicting densitometric percentages of HisMyc-NE81ΔNLSΔCLIM in supernatants and pellets obtained at the indicated salt conditions (low protein concentration = 0.2 mg/mL protein). (**D**) Percentages of HisMyc-NE81ΔNLSΔCLIM in supernatants and pellets obtained at the indicated salt and pH conditions (low protein concentration = 0.2 mg/mL protein). Key: +++ most protein in supernatant, + around half of protein in supernatant, -- most of protein in pellet.

For affinity purifications, HisMyc-NE81ΔNLSΔCLIM protein clusters needed to be solubilized. From in vitro experiments with mammalian and *C. elegans* lamin, it was known that high-salt conditions promote disassembly of filaments, while low-salt conditions favor their assembly [40,44]. Thus, we used high-salt conditions for detergent-free mechanical cell lysis and sedimentation of nuclei. The supernatant was then used for affinity chromatography with Ni-NTA beads. Elution with 250 mM imidazole at high-salt conditions led to a clear peak fraction containing the HisMyc-NE81ΔNLSΔCLIM protein. As assessed by comparison of Coomassie protein stainings and anti-myc Western blot analysis, this peak fraction no longer contained relevant amounts of contaminating proteins (Figure 5A). Therefore, this fraction was directly used for assembly/disassembly experiments. The protein was dialyzed into Tris buffers at varying NaCl concentrations. After centrifugation of the dialyzed protein at 17,000× *g*, the protein content in pellets and supernatants was analyzed by Western blotting. As expected, protein amounts in supernatants increased with increasing salt concentration, indicating that low-salt conditions promote assembly of filaments and vice versa (Figure 5B,C). This effect was stronger at lower protein concentrations (<0.2 mg/mL; Figure 5B). When no NaCl was added (0 M NaCl condition), more than 80% of the HisMyc-NE81ΔNLSΔCLIM protein was found in the pellet, whereas, at high-salt conditions (0.3 M NaCl), only ~30% was found in the pellet (Figure 5C,D). Higher pH (9.0 instead of 8.0) favored the solubility of HisMyc-NE81ΔNLSΔCLIM, an effect also seen with *C. elegans* lamin [40] (Figure 5D). At higher protein concentrations (>0.4 mg/mL), the purified protein required higher salt concentrations to solubilize, i.e., it exhibited a higher tendency to remain in the assembled state (Figure 5B). This effect was partially compensated for by increasing the NaCl concentration up to 0.5 M (Figure 5B). 

**Figure 6:**
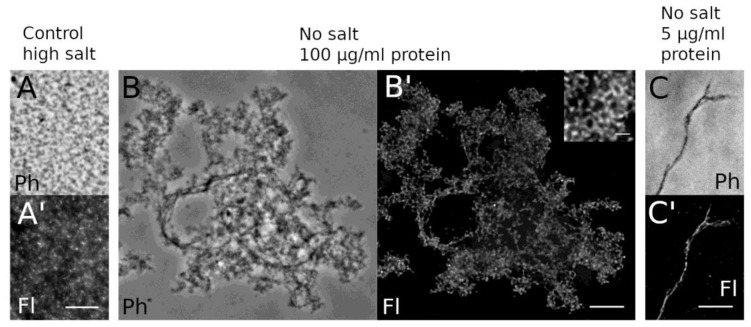
Light microscopy reveals reticular HisMyc-NE81ΔNLSΔCLIM assemblies. Purified protein was fixed with formaldehyde and stained with anti-NE81/anti-rabbit-AlexaFluor 488. Phase-contrast (**A**–**C**; Ph) and fluorescence images (**A**–**C′**; Fl) are shown. (**A**,**A′**) At high NaCl concentration, no clear assemblies are visible, whereas the protein forms reticular assemblies at no-salt conditions and high protein concentration (**B**,**B′**). At low protein concentration (**C**,**C′**), individual filamentous structures become apparent. Scale bars = 5 µm (inset = 1 µm).

**Figure 7:**
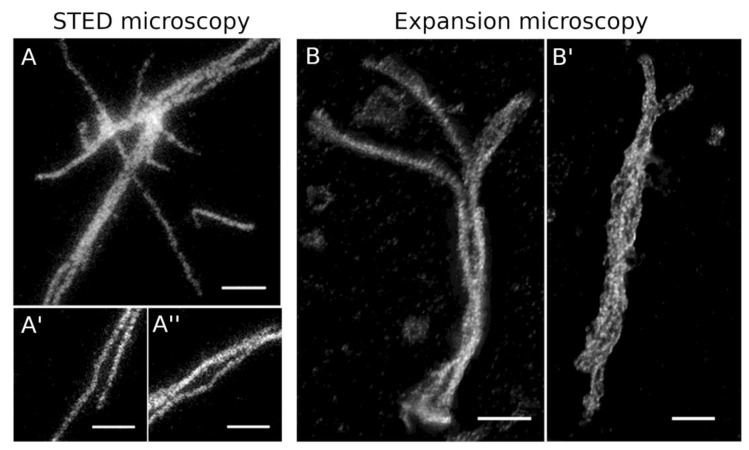
Super-resolution fluorescence microscopy of HisMyc-NE81ΔNLSΔCLIM filaments. (**A**–**A′′**) stimulated emission depletion (STED) microscopy: assemblies formed at low protein concentration (5 µg/mL) were stained with anti-NE81/anti-rabbit-Atto 647N. Scale bars = 1 µm. (**B**,**B′**) Expansion microscopy with deconvolution, 3.5-fold expanded; assemblies formed at 0.3 mg/mL (**B**) and 5 µg/mL (**B′**) were stained with anti-NE81/anti-rabbit-AlexaFluor 488. Maximum-intensity projections are presented. Refer to supplemental videos 1 and 2 to get a three-dimensional impression. The scale bar (2 µm) refers to the original size of the specimen.

For this reason, the purification buffer generally contained 0.5 M NaCl. A denaturation/renaturation step as employed in similar experiments with metazoan lamins [40,45] did not improve our results. For a quick structural analysis, the putative protein assemblies formed under low/no-salt conditions were concentrated by centrifugation onto coverslips, stained with anti-myc followed by AlexaFluor 488-conjugated secondary antibodies, and analyzed by phase-contrast and wide-field deconvolution microscopy. The latter achieves an optical resolution of ~170 nm [46]. At higher protein concentrations of ~100 µg/mL, HisMyc-NE81ΔNLSΔCLIM formed large reticular structures, while single filaments were detectable at lower concentrations of 10 µg/mL or less (Figure 6). To reach a higher resolution, we performed STED microscopy and expansion microscopy (ExM) according to the protocols published by the Boyden and Vaughan groups [29,30]. Both methods yielded comparable results and clearly showed that HisMyc-NE81ΔNLSΔCLIM is capable of forming filamentous assemblies (Figure 7). 

**Figure 8:**
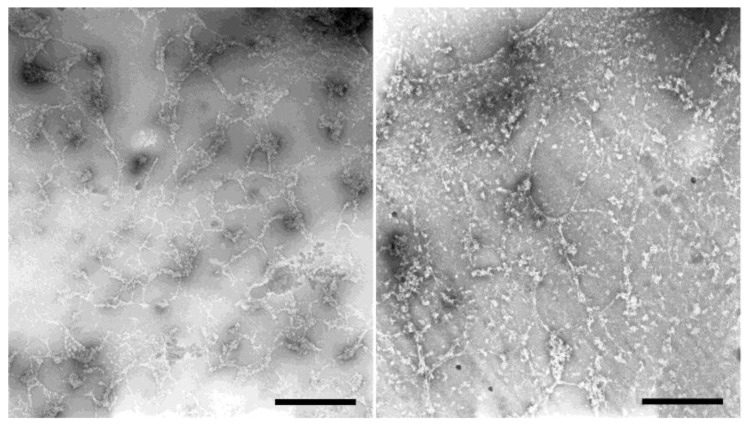
Reticular HisMyc-NE81ΔNLSΔCLIM filament networks visualized by negative-staining transmission electron microscopy. Two representative examples are shown. Scale bar = 1 µm.

As all these light microscopic methods have an optical resolution too low to resolve the filaments seen in our feSEM images or the diameters of intermediate filaments, it made no sense to evaluate filament thickness in these light microscopic images. However, if ExM images were deconvolved, a closer look revealed that individual filaments were closely aligned in parallel arrangements of two to four filaments (Figure 7B, B’, supplemental videos 1 and 2). Within these arrangements, filaments had a distance between each other of 150 ± 21 nm (mean ± SD, *n* = 20; referring to the original size; expansion factor 3.5). To gain further insight into the thickness of in vitro polymerized filaments, we performed negative-staining TEM analysis of HisMyc-NE81ΔNLSΔCLIM filaments. As expected from the light microscopic appearance (Figure 6B, inset), the resulting TEM images revealed filamentous structures in reticular arrangements (Figure 8). Filament thickness varied with a mean size of 13.2 ± 4.2 nm (mean ± SD, *n* = 50), i.e., thicker than the filaments formed at the surface of *Xenopus* nuclear membranes.

## 4. Discussion

In this work, we provide, for the first time, ultrastructural data of lamin assemblies in a non-metazoan organism. The high similarity of their appearance, compared to those obtained applying similar methods to various metazoan lamin isoforms, clearly underscores the evolutionary relationship of NE81 to lamins as proposed earlier [12]. Thus, we suggest the use of the term “lamin” also for NE81, instead of the previously used term “lamin-like protein”. Our major goal in this work was to examine whether NE81 is capable of forming filamentous structures at all. To facilitate protein isolation and detection, we used an NE81 variant carrying a small 8×HisMyc-tag. Our results strongly suggest that this tag does not interfere with NE81 assembly and function. Moreover, to avoid chromatin contamination and to allow protein isolation from the cytosol, we opted for an NE81 variant lacking both a CaaX-box and a functional NLS. Earlier experiments with GFP-NE81 and the behavior of HisMyc-NE81ΔNLSΔCLIM clusters in this work proved that these NLS and CaaX-box mutations did not compromise the capacity of cell-cycle-dependent assembly/disassembly of the protein. In feSEM images, FLAG-NE81 formed filamentous structures with an overall appearance highly reminiscent to that of *Xenopus* lamin B2 in the same assay. NE81 filament thickness was slightly higher than that measured with the same instrument in an analogous preparation of lamin B2 [31]. In negatively stained TEM images, filaments appeared even thicker. However, in this instance, it cannot be excluded that the difference in thickness (8.5 nm vs. 13.2 nm) was caused by the different NE81 variants used (FLAG-NE81 vs. HisMyc-NE81ΔNLSΔCLIM), or was solely based on different methodology or inaccuracies in scale calibration. Thus, we cannot make predictions of the number of protofilaments assembling into native NE81 filaments. The exact details of the structural organization of lamin filaments were investigated for decades and still remain a controversial matter. Aebi and co-workers were the first to successfully analyze lamin filament structure in *Xenopus* oocyte nuclei prepared for freeze-dried metal-shadowed electron microscopy [44]. In this specimen, lamin appeared to constitute highly ordered, orthogonally arranged filaments with a thickness of ~10.5 nm and a spacing of ~52 nm. This led to the conclusion that lamin filaments form similar 10-nm filaments as cytosolic IFs, which are composed of eight protofilaments, as in the case of vimentin. Yet, in *Xenopus* oocyte nuclei, these structures consist almost exclusively of the embryonic B-type lamin LIII, which is absent in most differentiated cells. When somatic lamins B1, B2, and A were expressed in *Xenopus* oocytes and the resulting assemblies were analyzed by feSEM, it appeared that B-type lamins formed 10-nm filaments arranged in a two-dimensional lattice, albeit at a narrower spacing between filaments than measured for lamin LIII. A-type lamins formed even thicker filaments arranged in a more irregular, three-dimensional pattern [31,38]. In cryo-EM images *C. elegans* lamin appeared as heterogeneous filaments of only 4–6 nm [47], indicating that lamins may also assemble into filaments thinner than the usual ~10 nm of cytosolic IFs. Recently, super-resolution light microscopy techniques opened a path to study fluorescent A- and B-type lamin networks in mouse adult and embryonic fibroblasts (MAFs and MEFs, respectively) [48,49,50,51]. These studies revealed that A- and B-type lamins assemble into distinct networks at the nuclear envelope, albeit lacking regular orthogonal arrangements. Due to the resolution limit, filament thickness could not be evaluated in these light microscopic studies. Yet, very recently, Medalia and co-workers succeeded in employing cryo-EM to disclose the ultrastructure of the lamin network in MEFs carrying a knockout of the cytosolic IF vimentin [52,53]. In these cells, lamins formed filaments of only ~3.5 nm, i.e., about the size of a typical IF protofilament, with a relatively irregular arrangement beneath the inner nuclear membrane. Successful in vitro experiments were mainly undertaken in the *C. elegans* model, as this was the only system to allow the formation of bona fide filaments starting from purified recombinant, bacterially expressed lamin [45]. Cryo-EM of these filaments revealed paracrystals and filaments composed of three to four protofilaments [54], confirming the feSEM data (see above) that already indicated thinner filaments with accordingly fewer protofilaments [47]. In in vitro experiments, in the presence of calcium, lamins showed a tendency to form large paracrystalline arrays with many filaments present and a thickness of ~100 nm [39]. The capacity of forming such large paracrystals may also depend on the presence of the C-terminal Ig-fold within the LTD present in most lamins [45,52]. This fold is absent in the tunicate *Ciona* lamin, which forms relatively thin 5.4-nm filaments and poorer paracrystalline arrays. These arrays had a thickness around 50 nm and appeared to consist of two intertwined filamentous assemblies [45]. This is reminiscent of the intertwined filamentous arrangements observed in ExM images of *Dictyostelium* lamin NE81. Admittedly, the LTD is not highly conserved in NE81, but clearly present and readily detected by PROSITE (https://prosite.expasy.org) and HMMER [55]. Yet, our results may indicate that this domain does not adopt the three-dimensional shape of an Ig-domain in *Dictyostelium*.

Taken together, in contrast to cytosolic IFs, lamins appear to be capable of forming a variety of supramolecular structures, depending on the individual lamin isoforms, cell types, and organisms. While the thickness of the *Dictyostelium* lamin filaments was in the range of *Xenopus* lamin LIII, their irregular arrangement was more reminiscent of that reported in MEFs and MAFs [48,49,53]. In fact, the appearance of reticular NE81 networks resembles that published for lamins in MEFs by Shimi and co-workers [48].

## 5. Conclusions

*Dictyostelium* amoebae provide the only non-metazoan model organism so far, with a well-characterized nuclear envelope involving all relevant protein components known in higher cells. Lamin (NE81) [12], the LEM (LAP2, emerin, MAN1)-family protein Src1 [18], the LINC component Sun1 [56], an interacting kinesin (Kif9) [57], nuclear pore complex (NPC) components [58], and endosomal sorting complex required for transport (ESCRT) components [59,60,61] are all present. Given the high similarity of *Dictyostelium* nuclear lamina organization to human cells, the speed and low cost of cell culture, the ease of genetic manipulation, and great accessibility for microscopic analysis, these amoebae offer the most attractive non-metazoan experimental model to study the molecular and structural basis of laminopathies.

## Supplementary Materials

The following are available online: http://www.mdpi.com/2073-4409/8/2/162/s1. Video S1: Expansion microscopy with deconvolution of HisMyc-NE81∆NLS∆CLIM filaments, 3.5-fold expanded; assemblies were stained with anti-NE81/anti-rabbit-AlexaFluor 488. A stack of 27 images (*z*-distance 0.3 µm) is presented. The scale bar (2 µm) refers to the original size of the specimen. Video S2: Expansion microscopy with deconvolution of HisMyc-NE81∆NLS∆CLIM filaments, 3.5-fold expanded; assemblies were stained with anti-NE81/anti-rabbit-AlexaFluor 488. A 360 °C rotational 3D animation of the stack shown in video 1 is presented. The scale bar (2 µm) refers to the original size of the specimen.
